# Diagnostic performance of commercial IgM and IgG enzyme-linked immunoassays (ELISAs) for diagnosis of Zika virus infection

**DOI:** 10.1186/s12985-018-1015-6

**Published:** 2018-07-13

**Authors:** Mariana Kikuti, Laura B. Tauro, Patrícia S. S. Moreira, Gúbio S. Campos, Igor A. D. Paploski, Scott C. Weaver, Mitermayer G. Reis, Uriel Kitron, Guilherme S. Ribeiro

**Affiliations:** 1 Instituto Gonçalo Moniz, Fundação Oswaldo Cruz, Rua Waldemar Falcão, 121, Candeal, 40296-710, Salvador, BA Brazil; 2 Universidade Federal da Bahia, Salvador, Brazil; 3 Instituto Nacional de Medicina Tropical CONICET, Puerto Iguazu, Misiones Argentina; 4 University of Texas Medical Branch, Galveston, TX USA; 5 Emory University, Atlanta, GA USA

**Keywords:** Zika virus, Sensitivity and specificity, Enzyme-linked immunosorbent assay, Immunoglobulin M, Immunoglobulin G

## Abstract

**Background:**

Serologic detection of Zika virus (ZIKV) infections is challenging because of antigenic similarities among flaviviruses.

**Objective:**

To evaluate the sensitivity and specificity of commercial ZIKV IgM and IgG enzyme-linked immunoassay (ELISA) kits.

**Methods:**

We used sera from febrile patients with RT-PCR-confirmed ZIKV infection to determine sensitivity and sera from RT-PCR-confirmed dengue cases and blood donors, both of which were collected before ZIKV epidemics in Brazil (2009–2011 and 2013, respectively) to determine specificity.

**Results:**

The ZIKV IgM-ELISA positivity among RT-PCR ZIKV confirmed cases was 0.0% (0/14) and 12.5% (1/8) for acute- and convalescent-phase sera, respectively, while its specificity was 100.0% (58/58) and 98.3% (58/59) for acute- and convalescent-phase sera of dengue patients, and 100.0% (23/23) for blood donors. The ZIKV IgG-ELISA sensitivity was 100.0% (6/6) on convalescent-phase sera from RT-PCR confirmed ZIKV patients, while its specificity was 27.3% (15/55) on convalescent-phase sera from dengue patients and 45.0% (9/20) on blood donors’ sera. The ZIKV IgG-ELISA specificity among dengue confirmed cases was much greater among patients with primary dengue (92.3%; 12/13), compared to secondary dengue (7.1%; 3/42).

**Conclusions:**

In a setting of endemic dengue transmission, the ZIKV IgM-ELISA had high specificity, but poor sensitivity. In contrast, the ZIKV IgG-ELISA showed low specificity, particularly for patients previously exposed to dengue infections. This suggests that this ZIKV IgM-ELISA is not useful in confirming a diagnosis of ZIKV infection in suspected patients, whereas the IgG-ELISA is more suitable for ZIKV diagnosis among travelers, who reside in areas free of flavivirus transmission, rather than for serosurveys in dengue-endemic areas.

## Background

Zika virus (ZIKV) is an emerging mosquito-borne flavivirus. Majority of human infections evolve asymptomatically or cause mild and self-limited clinical manifestations. Non-specific signs and symptoms may include exanthema, low-grade fever, conjunctivitis, and arthralgia [1–3]. However, following ZIKV outbreaks in French Polynesia and Brazil, increase in cases of Guillain-Barré syndrome in adults and of congenital abnormalities in newborns were identified, and subsequent studies confirmed ZIKV infection during pregnancy as a cause of congenital neurological abnormalities [4, 5].

Due to the similarity in the initial clinical presentation between ZIKV infections and other infectious diseases, especially with other arboviral diseases that usually co-occur in tropical and sub-tropical regions, such as dengue (DENV) and chikungunya (CHIKV), clinical diagnosis is challenging, and often depends on laboratory confirmation. However, ZIKV laboratory diagnosis is difficult because viremia tends to be low [6]. In addition, ZIKV presents structural similarities with other flaviviruses, especially DENV, resulting in cross-reactive antibodies [1, 7]. Consequently, serological tests for ZIKV may cross-react with DENV, particularly when pre-existing immunity to DENV is present [7–10].

A correct differential diagnosis between ZIKV and other arboviral infections is critical to alert patients and clinicians for potentially evolving complications, as well as to guide appropriate supportive care (i.e. fluid replacement for severe dengue patients). In addition, an accurate ZIKV laboratory diagnosis can inform surveillance activities, which include detection and monitoring of virus circulation, estimation of disease burden, guiding preventive and control actions to interrupt virus transmission, and informing pregnant women on the risk of a gestational infection.

### Objectives

In this study, we evaluated the diagnostic sensitivity of commercial enzyme-linked immunoassays (ELISAs) (Euroimmun, Lübeck, Germany) for detection of ZIKV IgM and IgG antibodies in sera of febrile patients with ZIKV infections. We also assessed the specificity of these kits in sera of both dengue-confirmed patients and blood donors.

### Study design

#### Study site and design

This study was conducted and reported according to the STARD (Standards for Reporting of Diagnostic Accuracy) guideline [11]. The performance of the IgM- and IgG-ELISAs was evaluated using serum collections from clinical-epidemiological studies conducted in Salvador, northeast Brazil. Since DENV introduction in Salvador in 1995, the city has experienced periodic DENV epidemics [12]. Between 2011 and 2016, approximately 7000 dengue cases per year were reported in Salvador [13], but the actual disease burden has been estimated to be at least 12 times greater [14]. In early 2015, Salvador faced a large outbreak of acute exanthematous illness later attributed to ZIKV, with over 17,000 reported cases [3, 4].

Most of the serum samples used for our diagnostic test evaluation were obtained from an acute febrile illness (AFI) enhanced surveillance study that we conducted in a public emergency health unit of Salvador, between January 2009 and August 2016. Details were previously described [15]. Briefly, the surveillance study enrolled patients ≥5 years of age with reported fever or measured axillary temperature ≥ 37.8 °C of up to 7 days of duration. Self-reported data on days of symptoms and clinical characteristics were obtained from participants at enrollment.

We collected blood samples from participants at study enrollment (acute-phase sample) and ≥ 15 days post-enrollment (convalescent-phase sample). Sera were stored in aliquots at − 20 °C and − 70 °C for serologic and molecular testing, respectively. Acute-phase samples were tested for DENV by NS1- and IgM-ELISA (Panbio Dengue Early ELISA and Panbio Dengue IgM Capture, Panbio Diagnostics, Brisbane, Australia) and convalescent-phase samples by IgM-ELISA (Panbio Dengue IgM Capture, Panbio Diagnostics, Brisbane, Australia). Between 2009 and 2014, acute-phase samples that yielded a positive result in either ELISA underwent RNA extraction (Maxwell® 16 Total RNA Purification kit by Promega, Wisconsin, USA, or QIAmp® Viral RNA mini kit by Qiagen, Hilden, Germany) and RT-PCR (AccessQuick RT-PCR System, Promega, Madison,WI) for dengue typing [16]. Beginning in 2014, however, all acute-phase samples underwent RT-PCRs for DENV [16], ZIKV [17] and CHIKV [18]. Dengue IgG-ELISA (Panbio Dengue IgG Indirect ELISA, Panbio Diagnostics, Brisbane, Australia) was performed for all acute-phase serum samples of dengue-confirmed cases; those with a negative result were considered a primary dengue infection, whereas those with a positive result were considered secondary dengue infections.

Additionally, we obtained sera from presumably health volunteer blood donors at the State Blood Donation Center located in Salvador during December 2013. Collection of blood was performed after the regular screening process (which, among others, excludes donors reporting fever in the previous 15 days). Blood donors’ sera were stored in − 20° and subsequently tested for DENV with the same NS1-, IgM- and IgG-ELISA kits.

#### Sample selection

To determine the sensitivity of the ZIKV IgM- and IgG-ELISA (Anti-Zika Virus IgM ELISA and Anti-Zika Virus IgG ELISA, EUROIMMUN, Lübeck, Germany), we tested the available sera (14 acute-phase and 8 convalescent-phase serum samples) for the 14 patients enrolled during the surveillance study who had a ZIKV infection confirmed by RT-PCR (all of them recruited between May and July, 2015). To determine the specificity of the ZIKV IgM- and IgG-ELISA in a group of confirmed dengue patients, we selected from RT-PCR-positive dengue cases detected during the AFI surveillance study the first 20 cases positive for each DENV serotype. We selected the first 20 confirmed cases of each DENV type to ensure that there was no recognized ZIKV transmission in Salvador at the time, such that there was limited chance of a prior ZIKV infection. The selected DENV1-confirmed patients were detected from April 2009 to May 2011; the DENV2 from February 2009 to February 2010; and DENV4 from October 2010 to April 2011. DENV-3 patients were not selected for testing because we confirmed only a very small number of such cases during the study period. To augment the confidence that these DENV patients did not have a concomitant ZIKV infection, we also performed ZIKV RT-PCR on their acute-phase serum samples. We also determined the specificity of the ZIKV IgM- and IgG-ELISA in randomly selected sera from 23 blood donors. The initially planned number of samples from DENV-confirmed patients and blood donors (143 samples: 60 DENV acute- and convalescent-phase samples each and 23 blood donor samples) was defined to provide 95% confidence for an estimated specificity of 95% and a precision of ~ 3.5%.

#### Zika IgM- and IgG-ELISA testing

The ZIKV IgM-ELISA (El 2668–9601 M) and ZIKV IgG-ELISA (El 2668–9601 G) by Euroimmun (Lübeck, Germany) were performed according to the manufacturer’s instructions for semiquantitative interpretations. Although assay performers were not blinded to group identification of tested samples (ZIKV, DENV, or blood donors), reading was performed by automated microplate reader (TECAN, Maennedorf, Switzerland). Positive and negative controls were provided by the test’s manufacturer.

#### Data analysis

The AFI patients with a RT-PCR-based diagnosis of ZIKV or DENV were characterized according to demographic characteristics (sex and age) and clinical manifestations. We calculated the accuracy of ZIKV IgM- and IgG-ELISA tests, with respective 95% confidence intervals, using the samples from RT-PCR-confirmed cases of ZIKV infection as the reference group of positives for sensitivity estimation, and the samples from RT-PCR-confirmed dengue cases, as well as blood donors as reference groups of negatives for specificity estimation. We also calculated the specificity among dengue-confirmed cases stratified by the infecting DENV type and by the type of infection (primary versus secondary). Samples with equivocal ELISA results were excluded from the accuracy analysis.

## Results

### Participants characteristics

The 14 RT-PCR-confirmed Zika cases had a median age of 22.5 years and 42.9% were male. The most frequent clinical manifestations were headache (92.9%), myalgia (85.7%), retro-orbital pain (71.4%), rash (71.4%), and pruritus (71.4%) (Table 1). Median time between symptoms onset and acute- and convalescent-phase blood collection was 2.5 and 27 days, respectively (Table 1). The 60 RT-PCR-confirmed dengue cases had a median age of 18 years and 51.6% were male. The most frequent clinical manifestations were headache (96.7%), myalgia (76.7%), retro-orbital pain (56.7%), and arthralgia (46.7%) (Table 1). Contrasting with the Zika cases, only 10.0% of the dengue cases presented with rash. The median time between onset of symptoms and acute- and convalescent-phase blood collection was 2 and 27 days, respectively (Table 1). The RT-PCR-positive dengue cases were also positive by NS1-ELISA (10 patients), by NS1- and IgM-ELISA in the acute-phase sera (6 patients), by IgM-ELISA seroconversion between acute- and convalescent-phase sera (23 patients), and by NS1-ELISA and IgM-ELISA seroconversion (21 patients). Of the 60 RT-PCR-positive dengue cases, 47 had available serum to test by ZIKV RT-PCR and all of them (100.0%) were ZIKV negative.

**Table 1:** Demographics, clinical characteristics, and serum sample availability for 14 Zika and 60 dengue cases confirmed by RT-PCR

Characteristics	Zika cases (*N* = 14)	Dengue cases (*N* = 60)
	Number (%) or median (interquartile range)	
Demographic		
Male	6 (42.9)	31 (51.7)
Age	22.5 (15–41)	18 (10–34)
Clinical Manifestations		
Headache	13 (92.9)	58 (96.7)
Myalgia	12 (85.7)	46 (76.7)
Retro-orbital pain	10 (71.4)	34 (56.7)
Arthralgia	8 (57.1)	28 (46.7)
Vomit	2 (14.3)	12 (20.0)
Rash	10 (71.4)	6 (10.0)
Pruritus	10 (71.4)	NA
Prior dengue infection^a^		
No	1 (8.3)	13 (21.7)
Yes	11 (91.7)	47 (78.3)
Blood sample collected		
Acute-phase sample	14 (100)	60 (100)
Time between symptoms onset and sample collection	2.5 (2–4)	2 (1–3)
Convalescent-phase sample	8 (57.1)	60 (100)
Time between symptoms onset and sample collection	27 (18.5–43.5)	27 (19.5–57.5)
Sera tested by ZIKV ELISA^b^		
ZIKV IgM-ELISA in acute-phase sera	14 (100)	58 (96.7)
ZIKV IgM-ELISA in convalescent-phase sera	8 (57.1)	59 (98.3)
ZIKV IgG-ELISA in acute-phase sera	13 (92.9)	NT
ZIKV IgG-ELISA in convalescent-phase sera	8 (57.1)	60 (100)

NT = Not tested

^a^Prior dengue infection was determined by a positive result in the DENV IgG-ELISA performed in the acute-phase serum sample

^b^Some patients did not have their acute- or convalescent serum sample tested for ZIKV because of insufficient volume of sera

For the Zika and dengue cases, the blood sample availability for ZIKV IgM- and IgG-ELISAs testing are also summarized in Table 1. The median age for the 23 blood donors was 32 years and 69.6% were male. All blood donors tested negative by dengue NS1- and IgM-ELISA, and 20 (87.0%) tested positive by DENV IgG-ELISA.

### ZIKV IgM-ELISA performance

As expected, none of the 14 acute-phase samples of RT-PCR-confirmed Zika cases yielded a positive result in the ZIKV IgM-ELISA, whereas one of 8 convalescent-phase samples was positive (12.5% sensitivity; Table 2). Zika IgM-ELISA yielded a positive result in only one of the 140 non-Zika sera tested (99.3% specificity overall; Table 2). None of the blood donors and acute-phase sera from dengue cases tested positive in the ZIKV IgM-ELISA (100% specificity in both groups). Among the convalescent-phase sera from dengue cases, only one (from a patient with a primary infection with DENV-2) tested positive in the ZIKV IgM-ELISA (98.3% specificity).

**Table 2:** Performance of the Euroimmun ZIKV IgM-ELISA kit

Performance according to the type of serum samples	ZIKV IgM-ELISA result		% (95% CI)
Sensitivity	Positive	Negative	
Acute-phase samples of RT-PCR-confirmed Zika cases^a^	0	14	0.0 (0.0–23.2)
Convalescent-phase samples of RT-PCR-confirmed Zika cases	1	7	12.5 (0.3–52.7)
Specificity	Negative	Positive	
Acute-phase sample of RT-PCR-confirmed dengue cases	58	0	100.0 (93.8–100.0)
Samples of DENV-1 infected cases	19	0	100.0 (82.4–100.0)
Samples of DENV-2 infected cases	19	0	100.0 (82.4–100.0)
Samples of DENV-4 infected cases	20	0	100.0 (83.2–100.0)
Samples of primary dengue cases	13	0	100.0 (75.3–100.0)
Samples of secondary dengue cases	45	0	100.0 (92.1–100.0)
Convalescent-phase sample of RT-PCR-confirmed dengue cases	58	1	98.3 (90.9–100.0)
Samples of DENV-1 infected cases	20	0	100.0 (83.2–100.0)
Samples of DENV-2 infected cases	18	1	94.7 (74.0–99.9)
Samples of DENV-4 infected cases	20	0	100.0 (83.2–100.0)
Samples of primary dengue cases	12	1	92.3 (64.0–99.8)
Samples of secondary dengue cases	46	0	100.0 (92.3–100.0)
Blood donors samples	23	0	100.0 (85.2–100.0)

^a^Acute-phase serum samples were collected at a mean of 3 days post onset of symptoms. Thus, the ZIKV IgM-ELISA was not expected to be positive at that time

### Zika IgG-ELISA performance

Of the 14 RT-PCR-confirmed Zika cases, 13 had had their acute-phase sera tested with the ZIKV IgG-ELISA and 6 (46.1%) were positive, 4 (30.8%) negative, and 3 (23.1%) equivocal (Table 3). All the 8 convalescent-phase samples from RT-PCR-confirmed Zika cases tested with the ZIKV IgG-ELISA were positive, yielding 100% sensitivity (Table 3). Among convalescent-phase sera from confirmed dengue cases, ZIKV IgG-ELISA yielded positive results in 40 (66.7%) samples, negative results in 15 (25.0%) samples, and equivocal results in 5 (8.3%) samples. Among the 23 sera from blood donors, 11 (47.8%) were positive in the ZIKV IgG-ELISA, 9 (39.1%) were negatives, and 3 (13.0%) generated equivocal results.

**Table 3:** Performance of the Euroimmun ZIKV IgG-ELISA kit

Performance according to the type of serum samples	ZIKV IgG-ELISA result		% (95% CI)
Sensitivity	Positive	Negative	
Acute-phase samples of RT-PCR-confirmed Zika cases^a^	6	4	40.0 (12.2–73.8)
Convalescent-phase samples of RT-PCR-confirmed Zika cases	8	0	100.0 (63.1–100.0)
Specificity	Negative	Positive	
Convalescent-phase samples of RT-PCR-confirmed dengue cases^b^	15	40	27.3 (16.1–41.0)
Samples of DENV-1 infected cases	9	11	45.0 (23.1–68.5)
Samples of DENV-2 infected cases	5	13	27.8 (9.7–53.5)
Samples of DENV-4 infected cases	1	16	5.9 (0.2–28.7)
Samples of primary dengue cases	12	1	92.3 (64.0–99.8)
Samples of secondary dengue cases	3	39	7.1 (1.5–19.5)
Blood donors samples^c^	9	11	45.0 (23.1–68.5)

^a^Of the 14 acute-phase serum samples from RT-PCR confirmed Zika cases, 1 was not tested by the ZIKV IgG-ELISA because of insufficient sample volume and 3 presented equivocal results and were not included in this analysis

^b^Of the 60 convalescent-phase serum samples from dengue cases tested with the ZIKV IgG-ELISA, 5 presented equivocal results and were not included in this analysis

^c^Of the 23 serum samples from blood donors tested with the ZIKV IgG-ELISA, 3 presented equivocal results and were not included in this analysis

Overall, the specificity of the ZIKV IgG-ELISA for the 75 samples with valid results was 32.0%. The specificity for convalescent-phase samples of dengue patients was 27.3%, ranging for 5.9% in DENV-4 infections to 45.0% in DENV-1 infections (Table 3). The specificity was much lower for patients diagnosed secondary DENV infections (7.1%) compared to those with primary DENV infections (92.3%). Specificity of the Zika IgG-ELISA among blood donors was 45.0%. All 11 blood donors’ samples for which the ZIKV IgG-ELISA returned a positive result had anti-dengue IgG antibodies, as did 6 of the 9 blood donors samples that were negative on the ZIKV IgG-ELISA. The mean optical density value found on the DENV IgG-ELISA of blood donors with positive results on the ZIKV IgG-ELISA was 2.738 (standard deviation [SD]: 0.179, min. 2.307, max. 2.965), a value statistically greater than the mean found on blood donors with negative results on the ZIKV-IgG-ELISA (1.549, SD: 1.283, min. 0.036, max. 2.873; Wilcoxon rank-sum test *P* = 0.04).

## Discussion

In this study, we evaluated the diagnostic performance of commercial IgM- and IgG-ELISAs for serodiagnosis of Zika virus infection. We used a set of sera collected from AFI patients prospectively enrolled in an enhanced surveillance study designed to detect arboviral infections, rather from a reference laboratory serum sample collection. Thus, we were able to characterize the clinical manifestations of the patients providing the sera tested, and to evaluate the accuracy of the tests in a group of patients’ samples that represent a realistic epidemiologic situation. Of note, clinical manifestations of RT-PCR-confirmed Zika and dengue AFI patients were similar, with the exception of rash, which was most commonly reported among Zika patients, reinforcing the difficulties in clinically differentiating these arboviral infections.

We found a high specificity for the Euroimmun ZIKV IgM-ELISA among both dengue-infected and blood donor control groups. Specificity among dengue controls was not affected by infecting serotype or prior dengue infection status. However, the sensitivity of the ZIKV IgM-ELISA was low for either acute- or convalescent-phase serum samples of Zika febrile patients (0.0 and 12.5%, respectively). As the time elapsed between Zika symptoms onset and acute-phase serum collection was short (median 2.5 days), a low IgM detection in the acute-phase sample was expected. However, even for the convalescent-phase serum samples, collected on average 3–6 weeks after disease onset, the ZIKV IgM-ELISA yielded a low sensitivity. This low performance was also reported for RT-PCR-confirmed Zika samples from residents from other ZIKV- and DENV-endemic areas (Suriname, Dominican Republic and Colombia), where Euroimmun Zika IgM-ELISA detected 6 of the 19 evaluated samples collected ≥1 days post onset of symptoms (dpos) and 5 of 12 samples collected ≥6 dpos, yielding sensitivities of 31.6 and 41.7%, respectively [19]. Interestingly, the same study reported sensitivities of 87.5 and 100.0% among RT-PCR-confirmed Zika samples from returning travelers with ≥1 dpos and ≥ 6 dpos, respectively, suggesting that previous flavivirus infections may alter ZIKV antibody responses.

Although recent studies suggest a high specificity of this Zika IgG-ELISA against dengue infections in returning travelers [19–22], we have demonstrated an overall low performance in a dengue-endemic region. This may be due to a greater frequency of secondary DENV infections in the population we studied (78.3%), in which specificity was 7.1% contrasting with 92.3% in primary infections. The low specificity of the Zika IgG-ELISA among our blood donor control group may be also explained by the high frequency (87.0%) of DENV IgG antibodies observed in this group. We also found a large difference in the ZIKV IgG-ELISA specificity among DENV-confirmed samples according to DENV serotype, varying from 5.9% in DENV4-infected patients to 45.0% in DENV1-infected patients. This difference is likely explained by the prior dengue-immune status of these patients, since 100% of DENV4-infected patients had secondary infections, contrasting with 65% of DENV1-infected patients. Although ZIKV IgG antibodies were detected in all 8 convalescent-phase samples of Zika cases, the same samples were also positive for dengue IgG antibodies, making it impossible to exclude cross-reactions.

The main limitation of our study was the small number of ZIKV-confirmed cases. Technical difficulties in ZIKV diagnosis, especially due to the low viremia during infection and the cross-reactions with other circulating arboviruses in tropical and subtropical regions, has hampered composing large subsets of Zika cases’ samples to be used in diagnostic test accuracy studies. As another limitation, we did not perform ZIKV RT-PCR on the acute-phase samples of all dengue cases to ensure the absence of co-infection. Even though, sufficient serum was available to perform RT-PCR in 47 of the 60 dengue cases, and none of them yielded a positive result. Considering that our dengue case selection specifically included patients with infections that occurred prior to 2012, it is unlikely that they had a dengue and Zika co-infection or a prior ZIKV infection, because ZIKV introduction into Brazil was estimated to have occurred between late 2012 and early 2013 [23–25]. In addition, prior ZIKV infections in the study group of blood donors cannot be completely ruled out because their serum samples were obtained in December 2013; but, as the ZIKV spread in Salvador was clearly recognized during a large outbreak in 2015, it is improbable that these individuals had ZIKV infections before study enrollment.

Strengths of our study included the use of RT-PCR as a confirmation criterion for both Zika and dengue cases because as ZIKV and DENV transmission are endemic in our setting, a molecular diagnosis ensures a more accurate distinction between these flaviviruses compared to serologic diagnosis. In addition, the availability of paired sera provided a unique opportunity to evaluate the performance of commercial ZIKV IgM- and IgG-ELISAs in detection of antibodies in acute- and convalescent-phase samples.

## Conclusion

In conclusion, our study revealed a high specificity but low sensitivity of the Euroimmun ZIKV IgM-ELISA in an endemic region for both dengue and Zika, as well as a high specificity of the Euroimmun ZIKV IgG-ELISA in DENV primary infections contrasting with low specificity in DENV secondary infections. This suggests that this IgG-ELISA may be more suitable for Zika diagnosis in travelers returning from non-endemic areas than for serosurveys in endemic areas. Further studies with larger well-characterized samples are needed to assess the diagnostic accuracy not only of this test, but also of several others serological ZIKV assays.
